# PPM1D is a prognostic marker and therapeutic target in colorectal cancer

**DOI:** 10.3892/etm.2014.1762

**Published:** 2014-06-06

**Authors:** TIAN-SHU PENG, YONG-HENG HE, TIAN NIE, XIANG-DANG HU, HAI-YAN LU, JIAN YI, YUN-FEI SHUAI, MIN LUO

**Affiliations:** 1 Department of Anorectal Disease, The Second Affiliated Hospital of Hunan University of Chinese Medicine, Changsha, Hunan 410005, P.R. China; 2 Department of Blood and Oncology, The First Affiliated Hospital of Hunan University of Chinese Medicine, Changsha, Hunan 410007, P.R. China

**Keywords:** colorectal cancer, PPM1D, prognosis, biomarker

## Abstract

Protein phosphatase, Mg^2+^/Mn^2+^ dependent, 1D (PPM1D) has been associated with carcinogenesis. The present study investigated PPM1D expression as a potential biomarker in colorectal cancer (CRC). PPM1D expression was assessed using immunohistochemistry in 368 patients with CRC. The correlation between PPM1D expression, clinicopathological features and prognosis was analyzed. PPM1D small interfering (si)RNA-induced PPM1D silencing was performed in CRC cell lines to assess the effect of PPM1D on tumor cell proliferation and invasion *in vitro*. A total of 68.48% (252/368) of the CRC samples displayed high PPM1D expression. By contrast, only 9.24% (34/368) of the matched non-cancerous tissue samples exhibited high PPM1D expression. High PPM1D expression was correlated with node metastasis (P=0.0024), distant metastasis (P<0.001) and TNM stage (P=0.0016). Kaplan-Meier survival analysis revealed that patients with low PPM1D expression had significantly longer survival than those with high PPM1D expression (P=0.012). Moreover, multivariate analyses demonstrated that high PPM1D expression was an independent prognostic factor for overall survival (hazard ratio = 0.24; 95% confidence interval, 0.13–0.86; P=0.004). Furthermore, PPM1D gene silencing was found to significantly reduce the proliferation and invasion of CRC cells *in vitro*. These findings suggest a role for PPM1D as a prognostic marker and potential therapeutic target in CRC.

## Introduction

Colorectal cancer (CRC) is the third most common cancer in humans worldwide (1). It has recently been reported that CRC mortality accounts for ~9% of all cancer mortalities (2). The survival rate for CRC is higher at early stages following surgical resection; however, the long-term survival rate and prognosis for patients with CRC remain poor (3). Currently available cancer markers are not suitable for current clinical practice and require further investigation. Therefore, there is an urgent requirement for the discovery of novel targets and useful biomarkers for CRC prognosis.

Protein phosphatase, Mg^2+^/Mn^2+^ dependent, 1D (PPM1D) is a member of the protein phosphatase 2C family and is involved in a wide range of physiological functions, including cell signaling, apoptosis and the cell cycle (4). A growing body of evidence suggests that PPM1D is involved in tumorigenesis. PPM1D has been reported to be upregulated in human primary breast, ovarian and neuroblastoma tumors (5–7). Of note, PPM1D has been found to complement oncogenes during cellular transformation (8). However, the role of PPM1D in CRC is yet to be elucidated. The present study investigated the expression pattern and clinical significance of PPM1D in CRC, including the correlation of PPM1D with clinical outcome.

## Materials and methods

### Patients and samples

Based on tissue data availability, 368 cases of CRC, between 2003 and 2007, were included in the present study. Clinicopathological features of the patients with CRC are shown in Table I. Formalin-fixed paraffin-embedded tissues were collected from the Second and First Affiliated Hospitals of Hunan University of Chinese Medicine (Changsha, China). This study was approved by the Ethics Committees of Hunan University of Chinese Medicine. Informed consent was obtained from all participants and the study was performed in accordance with the Declaration of Helsinki. Follow-up data were obtained from medical records and direct communication with the patients or their relatives. The follow-up period was defined as the time from the date of surgery to the date of patient mortality or the final follow-up in January 2012.

### Histopathological evaluation and scoring

Paraffin-embedded serial sections of CRC specimens were analyzed for PPM1D protein expression using an anti-PPM1D antibody (Abcam, Cambridge, MA, USA), as described previously (9,10). Negative control sections were incubated with pre-immunized rabbit serum (Abcam).

Immunostaining was assessed by two independent, blinded pathologists and scored by multiplying the intensity of the staining by the percentage of stained cells. The staining intensity was graded on a scale of 0 to 3. The percentage of stained tumor cells was graded as follows: 0 (<5%), 1 (5–25%), 2 (26–50%), 3 (51–75%) and 4 (>75%). The final scores ranged from 0 to 12. For any subsequent analysis, scores between 0 and 4 were defined as low expression and those between 5 and 12 were defined as high expression (11). Inconsistent scores were re-evaluated by two pathologists until a consensus score was established.

### Quantitative polymerase chain reaction (qPCR) analysis

Total RNA was extracted from cells using an RNA extraction kit (Qiagen Co., Ltd., Shanghai, China) according to the manufacturer’s instructions. Total RNA was used for quantification using the One-Step RT-PCR kit (Invitrogen Life Technologies, Carslbad, CA, USA) in a Roche Light Cycler (Roche Diagnostics, Basel, Switzerland). The primer sequences used in the study were as follows: PPM1D, 5′-CAATTGGCCTTG TGCCTACT-3′ (forward) and 5′-TCTTTCGCTGTGAGGTTGTG-3′ (reverse); and β-actin, 5′-CCTGTACGCCAACACAGTGC-3′ (forward) and 5′-ATACTCCTGCTTGCTGATCC-3′ (reverse). The PCR cycling conditions consisted of a denaturation step at 95°C for 3 min, followed by 40 cycles of 95°C for 10 sec and 60°C for 1 min. Samples were normalized using β-actin mRNA expression and relative expression was calculated using the ^ΔΔ^Ct method.

### Western blot analysis

Cells were lysed in lysis buffer and protein concentrations were measured using a BCA protein assay kit (Qiagen Co., Ltd.). Total protein was separated using SDS-PAGE on a 12.5% gel and electroblotted onto polyvinylidene fluoride membranes (Millipore Corporation, Billerica, MA, USA). Membranes were immunoblotted overnight at 4°C with primary antibodies against human PPM1D (1:1,000 dilution; Sigma-Aldrich, St. Louis, MO, USA) or β-actin (1:2,000 dilution; Sigma-Aldrich). Following three washes with Tris-buffered saline containing Tween 20, membranes were incubated with horseradish peroxidase-conjugated IgG secondary antibodies (1:2,000 dilution; Santa Cruz Biotechnology, Inc., Santa Cruz, CA, USA) for 2 h. Immunoreactive signals were detected using an enhanced chemiluminescence detection reagent (Pierce Biotechnology, Rockford, IL, USA). Expression levels were quantified from the images using Quantity One^®^ (Bio-Rad, Hercules, CA, USA). All experiments were performed in triplicate.

### Cell culture and transfection

HCT-116, RKO and COLO-320 CRC cell lines were purchased from American Type Culture Collection (Rockville, MD, USA). Cells were maintained in RPMI-1640 in 5% CO_2_ at 37°C. PPM1D siRNA and scrambled siRNA were purchased from Invitrogen Life Technologies and transfected using Lipofectamine^®^ 2000 (Invitrogen Life Technologies) according to the manufacturer’s instructions.

### MTT assay

Cell viability was assessed using an MTT assay. Following transfection, cells were plated in 96-well plates and incubated for 24, 48 and 72 h. A total of 20 μl 5 mg/ml MTT (Sigma-Aldrich) was added to each corresponding test well and incubated for 4 h at 37°C. The supernatant was then discarded and 200 μl dimethyl sulfoxide was added to each well to dissolve the formazan. Optical density was assessed by measuring the absorbance of each well at 490 nm using a spectrophotometer (SpectraMax Plus384; Molecular Devices, Sunnyvale, CA, USA). All experiments were performed in triplicate.

### Cell invasion assay

At 12 h after transfection, the mixed medium containing Lipofectamine 2000 was discarded and cells were maintained in serum-free RPMI-1640 medium overnight. Cell invasion was then assessed using a 24-well Cell Invasion assay (BD Biosciences, San Jose, CA, USA) with 8 μm pores according to the manufacturer’s instructions. Cells were incubated for 72 h. The upper compartment was coated with 50 μg Matrigel™ (BD Biosciences) and the lower compartment was filled with medium containing 10% fetal calf serum as a chemoattractant. Spectrophotometry was conducted using a microtiter plate reader (SpectraMax Plus384; Molecular Devices) at 540 nm. All assays were performed in triplicate.

### Statistical analysis

All statistical analyses were performed using SPSS 16.0 software (SPSS, Inc, Chicago, IL, USA). The χ^2^ test was performed for categorical data. Kaplan-Meier estimates, log-rank tests and multivariate Cox proportional hazard regression models were performed for survival analyses. A value of P<0.05 was considered to indicate statistical significance.

## Results

### Correlation between PPM1D and clinicopathological variables

**Figure 1:**
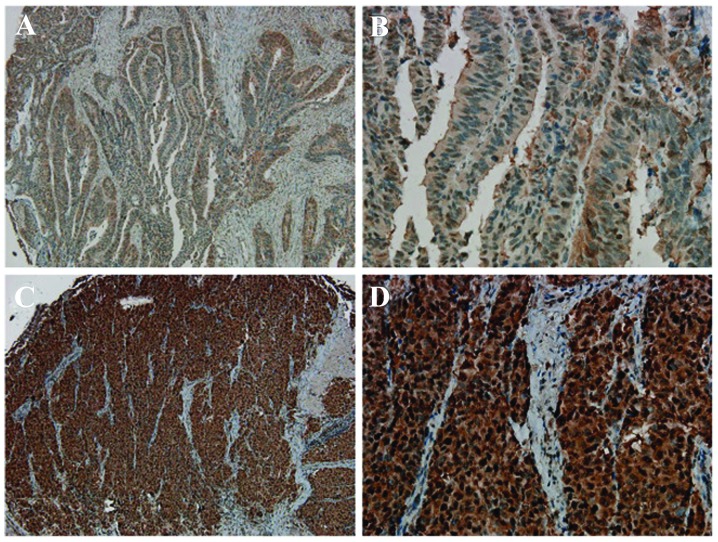
Immunohistochemical nuclear staining of PPM1D in colorectal cancer. Colorectal cancer samples were classified as having (A and B) low or (C and D) high PPM1D expression. Magnification: A and C, ×100; B and D, ×400. PPM1D, protein phosphatase, Mg^2+^/Mn^2+^ dependent, 1D.

PPM1D expression was analyzed in 368 CRC tissue samples and paired non-cancerous colorectal tissue samples. Among the 368 CRC tissues, 68.48% (252/368) of cases demonstrated high PPM1D expression, while only 9.24% (34/368) of the matched, non-cancerous colorectal tissue samples showed high PPM1D expression (P<0.001). Immunohistochemistry revealed a predominantly nuclear localization of PPM1D (Fig. 1) and showed that PPM1D expression was significantly higher in CRC tissue than in non-cancerous, normal colorectal tissue. In addition, significant differences in PPM1D expression were observed between tumors with node metastasis (P=0.0024), distant metastasis (P<0.001) and different TNM stages (P=0.0016; Table I). No significant correlation was observed between PPM1D expression and patient age or gender or tumor location or histology (Table I).

### PPM1D expression and survival in patients with CRC

**Figure 2:**
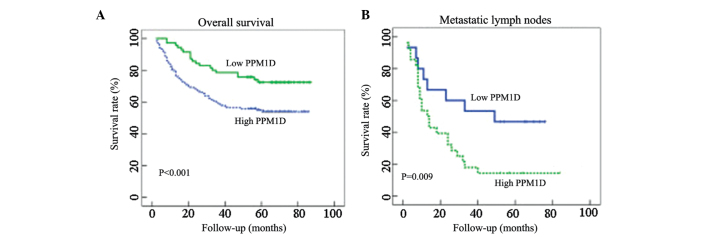
Kaplan-Meier curves showing PPM1D expression and overall survival in patients with CRC. (A) Patients with high PPM1D expression levels had poorer overall survival compared with patients with low PPM1D expression. Data are representative of 368 CRC tissues (P<0.001). (B) Patients with high PPM1D expression in metastatic lymph nodes had poorer overall survival than patients with low metastatic lymph node PPM1D levels. Data are representative of 74 metastatic lymph node tissues (P=0.009). PPM1D, protein phosphatase, Mg^2+^/Mn^2+^ dependent, 1D; CRC, colorectal cancer.

Kaplan-Meier survival analysis revealed that the patients with low PPM1D expression had significantly longer survival than those with high PPM1D expression (log-rank, P<0.001; Fig. 2A). The correlation between metastatic lymph node PPM1D expression and patient survival was also assessed. Kaplan-Meier analysis revealed that patients with low PPM1D expression in the metastatic lymph nodes had significantly longer overall survival than patients with high metastatic lymph node PPM1D expression (Fig. 2B, log-rank P=0.009).

Furthermore, multivariate analysis was performed using the Cox proportional hazards model. High PPM1D expression was identified to be a significant independent prognostic factor for OS (hazard ratio=0.24; 95% confidence interval, 0.13–0.86; P=0.004) as shown in Table II.

### PPM1D siRNA significantly reduces CRC cell proliferation and invasion

**Figure 3:**
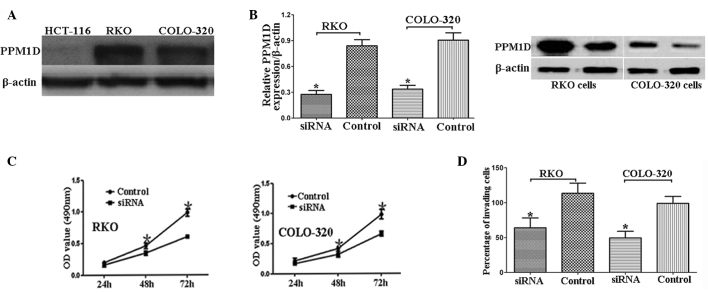
PPM1D siRNA significantly reduces proliferation and invasion in colorectal cancer cells. (A) PPM1D expression in HCT-116, RKO and COLO-320 cells detected using western blot analysis. (B) siRNA-induced PPM1D silencing confirmed using quantitative polymerase chain reaction and western blot analyses. (C) PPM1D siRNA inhibited the proliferation of RKO and COLO-320 cells. (D) PPM1D siRNA inhibited invasion by RKO and COLO-320 cells. Data are presented as the mean ± standard deviation of three independent experiments. ^*^P<0.05 for the difference between the two groups.

PPM1D expression was analyzed in three CRC cell lines: HCT-116, RKO and COLO-320. PPM1D expression was observed to be higher in RKO and COLO-320 cells than in HCT-116 cells (Fig. 3A). Based on this finding, RKO and COLO-320 cells were used for the subsequent functional analysis.

siRNA-induced PPM1D knockdown was confirmed using qPCR and western blot analyses (Fig. 3B). PPM1D siRNA was observed to significantly reduce proliferation and invasion in RKO and COLO-320 CRC cells (Fig. 3C and D).

## Discussion

The present study investigated the correlation between PPM1D expression, survival and clinical and pathological features in patients with CRC. Significant correlations were observed between PPM1D expression, metastasis, TNM stage and mortality. Furthermore, these correlations were found to be independent of other patient characteristics. These findings indicate that high PPM1D expression may be a useful prognostic marker for CRC.

PPM1D has been reported to be upregulated in neuroblastoma, as well as pancreatic, lung, bladder, liver, ovarian and breast cancer (6,7,12,13). However, the role of PPM1D in CRC is yet to be elucidated. In the present study, immunohistochemistry revealed that PPM1D was upregulated in CRC tissues compared with the levels in paired non-cancerous tissues. In human breast tissue, PPM1D overexpression has been found to contribute to malignant progression through inactivating wild-type p53 and p38 mitogen-activated protein kinase, as well as through decreasing p16 protein expression (14). PPM1D has also been shown to be a prognostic marker in patients with lung adenocarcinoma (15). Furthermore, high PPM1D expression has been reported to be correlated with poor prognosis in patients with pancreatic neuroendocrine tumors and medulloblastoma (16,17). In the present study, patients with CRC with high PPM1D expression were observed to have a worse outcome than those with low PPM1D expression. Furthermore, multivariate analysis suggested that high PPM1D expression was an independent prognostic factor for patients with CRC. *In vitro* experiments in CRC cells were also performed and revealed that PPM1D siRNA significantly inhibited CRC cell proliferation and invasion. These findings indicate that PPM1D may not only be a prognostic marker, but also a potential therapeutic target.

In conclusion, PPM1D may be a prognostic biomarker for CRC and its high expression is associated with poorer prognosis. Further investigations are required to validate the findings of the present study and to elucidate the underlying mechanisms through which PPM1D affects CRC.

## Figures and Tables

**Table I:** Clinicopathological characteristics and PPM1D expression in patients with colorectal cancer.

		PPM1D expression		
			
Characteristic	n	Low	High	P-value
Total	368	116	252	
Age (years)				0.371
<60	144	52	92	
≥60	224	64	160	
Gender				0.49
Male	208	72	136	
Female	160	44	116	
Tumor location				0.875
Right colon	80	24	56	
Left colon	92	32	60	
Rectum	196	60	136	
Histology (differentiation)				0.098
Well	172	48	124	
Moderate	136	56	80	
Poor	60	12	48	
Node metastasis				0.0024
N0	160	72	88	
N1–3	208	44	164	
Distant metastasis				<0.001
No	288	112	176	
Yes	80	4	76	
TNM stage				0.0016
I	28	12	16	
II	112	24	88	
III	148	72	76	
IV	80	8	72	

PPM1D, protein phosphatase, Mg^2+^/Mn^2+^ dependent, 1D.

**Table II:** Uni- and multivariate analyses of survival in patients with colorectal cancer.

	Overall survival			
	
	Univariate		Multivariate	
		
Variable	HR (95% CI)	P-value	HR (95% CI)	P-value
Age (years)				
<65	1			
≥65	1.003 (0.61, 1.58)	0.73		
Gender				
Male	1			
Female	0.74 (0.43, 1.19)	0.46		
TNM stage				
I	1		1	
II	0.36 (0.07, 1.51)	0.18	0.96 (0.21, 4.14)	0.96
III	0.09 (0.02, 0.41)	0.001	0.47 (0.06, 1.18)	0.08
IV	0.34 (0.19, 0.56)	<0.001	0.19 (0.10, 0.43)	<0.001
Differentiation				
Well	1			
Moderate/poor	0.14 (0.07, 0.235)	0.098		
PPM1D status				
Low expression	1		1	
High expression	0.04 (0.01, 0.09)	<0.001	0.24 (0.13, 0.86)	0.004

HR, Hazard ratio; CI, confidence interval; PPM1D, protein phosphatase, Mg^2+^/Mn^2+^ dependent, 1D.
